# mTOR-dependent alterations of Kv1.1 subunit expression in the neuronal subset-specific *Pten* knockout mouse model of cortical dysplasia with epilepsy

**DOI:** 10.1038/s41598-018-21656-8

**Published:** 2018-02-23

**Authors:** Lena H. Nguyen, Anne E. Anderson

**Affiliations:** 1 Department of Neuroscience, Baylor College of Medicine, Houston, TX USA; 2 The Jan and Dan Duncan Neurological Research Institute, Texas Children’s Hospital, Houston, TX USA; 3 The Gordon and Mary Cain Pediatric Neurology Research Foundation Laboratories, Texas Children’s Hospital, Houston, TX USA; 4 Department of Pediatrics, Baylor College of Medicine, Houston, TX USA; 5 Department of Neurology, Baylor College of Medicine, Houston, TX USA

## Abstract

Cortical dysplasia (CD) is a common cause for intractable epilepsy. Hyperactivation of the mechanistic target of rapamycin (mTOR) pathway has been implicated in CD; however, the mechanisms by which mTOR hyperactivation contribute to the epilepsy phenotype remain elusive. Here, we investigated whether constitutive mTOR hyperactivation in the hippocampus is associated with altered voltage-gated ion channel expression in the neuronal subset-specific *Pten* knockout (NS-*Pten* KO) mouse model of CD with epilepsy. We found that the protein levels of Kv1.1, but not Kv1.2, Kv1.4, or Kvβ2, potassium channel subunits were increased, along with altered Kv1.1 distribution, within the hippocampus of NS-*Pten* KO mice. The aberrant Kv1.1 protein levels were present in young adult (≥postnatal week 6) but not juvenile (≤postnatal week 4) NS-*Pten* KO mice. No changes in hippocampal Kv1.1 mRNA levels were found between NS-*Pten* KO and WT mice. Interestingly, mTOR inhibition with rapamycin treatment at early and late stages of the pathology normalized Kv1.1 protein levels in NS-*Pten* KO mice to WT levels. Together, these studies demonstrate altered Kv1.1 protein expression in association with mTOR hyperactivation in NS-*Pten* KO mice and suggest a role for mTOR signaling in the modulation of voltage-gated ion channel expression in this model.

## Introduction

Cortical dysplasia (CD) and other malformations of cortical development (MCDs) are highly associated with severe and intractable epilepsy, a condition characterized by spontaneous, recurrent seizures, and account for the first and third most common cause for epilepsy surgery in children and adults, respectively^1–3^. Aberrant hyperactivation of the mechanistic target of rapamycin (mTOR) signaling pathway, due to genetic mutations in the components of the pathway, has been implicated in CD and other MCDs^1,4^. However, the molecular alterations associated with mTOR hyperactivation and candidate mechanisms by which dysregulation of this pathway contribute to epilepsy in these disorders are not well understood.

The mTOR pathway is an evolutionarily conserved intracellular signaling pathway that is regulated upstream by phosphatidylinositol 3-kinase (PI3K)-Akt signaling^5^. The mTOR molecule itself is a serine/threonine kinase that exerts its functions through two distinct complexes, mTOR complex 1 and 2 (mTORC1 and 2, respectively), which are distinguished by their associated proteins and sensitivity to the inhibitor rapamycin. mTORC1 consists of mTOR associated with raptor, PRAS40, and mLST8, and is a rapamycin-sensitive complex involved in the regulation of protein synthesis, lipid synthesis, autophagy, energy metabolism, and lysosome biogenesis^5^. mTORC2 consists of mTOR associated with rictor, Sin1, and mLST8, and is a rapamycin-insensitive complex largely known for its role in regulating actin dynamics^5–7^. mTORC2 signaling is insensitive to acute rapamycin exposure but can be suppressed by prolonged rapamycin treatment^8,9^. One central and highly conserved role of mTOR signaling is to mediate protein synthesis through mRNA translational control^5^. Thus, it has been hypothesized that mTOR dysregulation in pathological conditions leads to altered protein expression contributing to the disease^10,11^. However, the molecular changes associated with genetic models of mTOR hyperactivity and epilepsy are not well defined.

Voltage-gated ion channels play critical roles in the regulation of neuronal excitability, and dysregulation of ion channel function, localization, and expression have been associated with hyperexcitability, increased seizure susceptibility, and epilepsy in humans and animal models^12,13^. Interestingly, physiological activation of mTOR has been found to suppress local translation of dendritic Kv1.1 potassium channels in hippocampal neurons^14^. Additionally, mice with neuronal-specific deletion of phosphatase and tensin homolog (Pten), a negative regulator of mTOR whose loss-of-function leads to mTOR hyperactivation, show decreased expression of Kv4.2 potassium channels^15^. Thus, mTOR dysregulation could potentially contribute to hyperexcitability and recurrent seizures by altering the protein expression of voltage-gated potassium channels.

Kv1 voltage-gated potassium channels consist of four integral pore-forming, voltage-sensing α subunits and four cytoplasmic auxiliary β subunits that help modulate channel function, localization, and surface expression^16,17^. Kv1 subunits co-assemble into homo- or heterotetrameric channel complexes giving rise to a remarkable number of channels with diverse functions^17^. In the hippocampus, the predominant α subunits, Kv1.1, Kv1.2, and Kv1.4, are highly expressed in the hilus and the middle third molecular layer of the dentate gyrus (DG), as well as in Schaffer collateral and mossy fiber axons, with subcellular localization in the axon initial segment (AIS), juxtaparanodes, and axon preterminals^17,18^. Kv1 channels regulate action potential duration, initiation, and propagation as well as membrane repolarization and neurotransmitter release, and dysfunction in Kv1 channels has been linked to epilepsy^12,13,18,19^.

Given that mTOR signaling plays an important role in protein synthesis, including translational control of Kv1.1, and that changes in Kv1 channel expression are associated with epilepsy, we investigated whether constitutive mTOR hyperactivation is associated with alterations in Kv1 channel expression in a previously characterized neuronal subset-specific *Pten* knockout mouse model of CD with epilepsy^20^. Insights into the molecular consequences of mTOR dysregulation are crucial to a better understanding of the underlying disease mechanisms and the development of novel anti-epileptic treatment strategies.

## Results

### PI3K-Akt-mTOR signaling is hyperactivated in the hippocampus of NS-*Pten* KO mice

**Figure 1:**
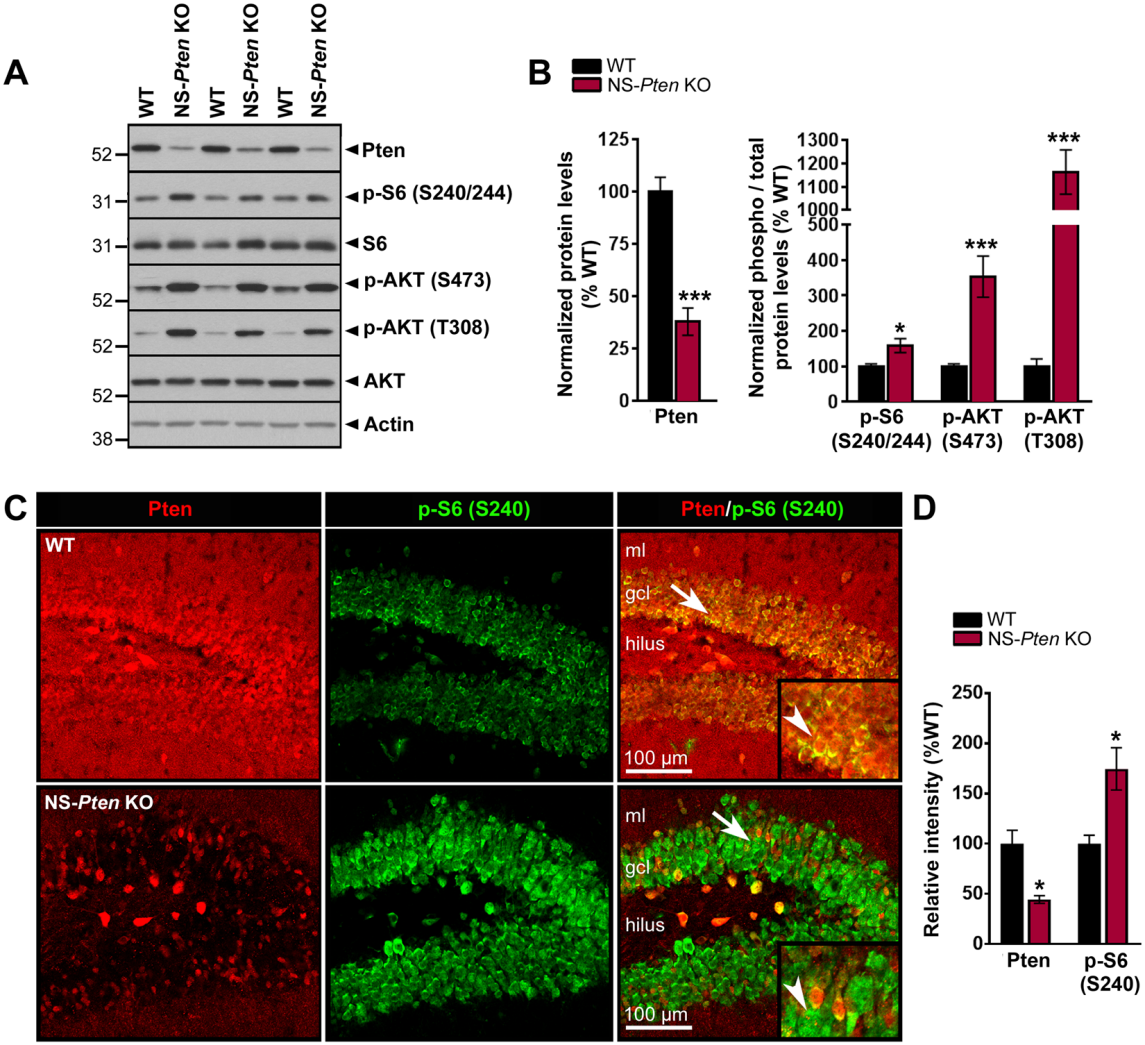
PI3K-Akt-mTOR signaling is hyperactivated in the hippocampus of NS-*Pten* KO mice. (**A**) Representative western blots from whole hippocampal homogenates from 6 week-old WT and NS-*Pten* KO mice probed with antibodies against Pten, p-S6 (S240/244; marker of mTORC1 activation), S6, p-AKT (S473; marker of mTORC2 activation), p-AKT (T308; marker of PI3K activation), AKT, and actin are shown. Full-length blots are presented in Supplemental Fig. 2. (**B**) Quantification of the western blots showed that Pten protein levels were significantly reduced while p-S6 (S240/244), p-AKT (S473), and p-AKT (T308) protein levels were significantly increased in NS-*Pten* KO compared to WT mice. n = 8 mice per group; *p < 0.05, ***p < 0.001 by Student’s t-test; errors bars are ± SEM. (**C**) Representative single optical sections of Pten and p-S6 (S240) co-immunostaining in 6 week-old WT and NS-*Pten* KO DG are shown. Arrows point to the areas that are enlarged in the insets. Arrowheads in the insets point to Pten-positive cells with basal p-S6 (S240) staining in WT DG and Pten-negative cells with elevated p-S6 (S240) staining in NS-*Pten* KO DG. (**D**) Quantification of Pten and p-S6 (S240) immunostaining intensity in the DG granule cell layer show significantly decreased Pten intensity and significantly increased p-S6 intensity in NS-*Pten* KO compared to WT mice. n = 3 mice per group. *p < 0.05 by Student’s t-test; errors bars are ± SEM. *gcl, granule cell layer; ml, molecular layer*.

Previous studies have shown selective deletion of Pten in the hippocampus and cortex of NS-*Pten* KO mice^20–22^. Since Pten is a negative regulator of mTOR that suppresses mTOR activity by counteracting the upstream PI3K-Akt pathway, loss of Pten is expected to increase PI3K-Akt-mTOR activation^23^. For these studies, we focused on changes associated with Pten loss in the hippocampus. We first quantified the loss of Pten proteins and confirmed PI3K-Akt-mTOR hyperactivation in NS-*Pten* KO hippocampi by western blotting. Pten protein levels were significantly decreased by more than two-fold in the hippocampi from 6 week-old NS-*Pten* KO compared to WT mice (p < 0.001 by Student’s t-test) (Fig. 1A,B). The resulting effects of hippocampal Pten loss on PI3K-Akt-mTOR activity were assessed by evaluating phosphorylation levels of S6 ribosomal protein at the S240/244 site [p-S6 (S240/244); a marker of mTORC1 activation]^10^, AKT at the S473 site [p-AKT (S473); a marker of mTORC2 activation]^24^, and AKT at the T308 site [p-AKT (T308); a marker of PI3K activation]^25^. We found significant increases in the protein levels of p-S6 (S240/244) (p < 0.01 by Student’s t-test), p-AKT (S473) (p < 0.001 by Student’s t-test), and p-AKT (T308) (p < 0.001 by Student’s t-test) in the hippocampus of NS-*Pten* KO compared to WT mice, indicating hyperactivation of the PI3K-Akt-mTOR pathway (Fig. 1A,B). Further evaluation using immunostaining for Pten revealed, in agreement with previous reports^21,22^, marked loss of Pten staining in the DG granule cell layer of NS-*Pten* KO compared to WT mice (Fig. 1C). Additionally, Pten staining in WT DG granule cells was associated with weaker basal p-S6 (S240) staining, while loss of Pten staining in NS-*Pten* KO DG granule cells was associated with stronger p-S6 (S240) staining (Fig. 1C, insets). Quantification of the staining intensities revealed a significant decrease in Pten (p < 0.05 by Student’s t-test) and a significant increase in p-S6 (S240) (p < 0.05 by Student’s t-test) intensity in NS-*Pten* KO DG compared to WT DG (Fig. 1D). Taken together, these results confirm the presence of Pten loss and hyperactivation of PI3K-Akt-mTOR signaling in the hippocampus of NS-*Pten* KO mice.

### Hippocampal Kv1.1, but not Kv1.2, Kv1.4, or Kvβ2, protein levels are increased in NS-*Pten* KO mice

**Figure 2:**
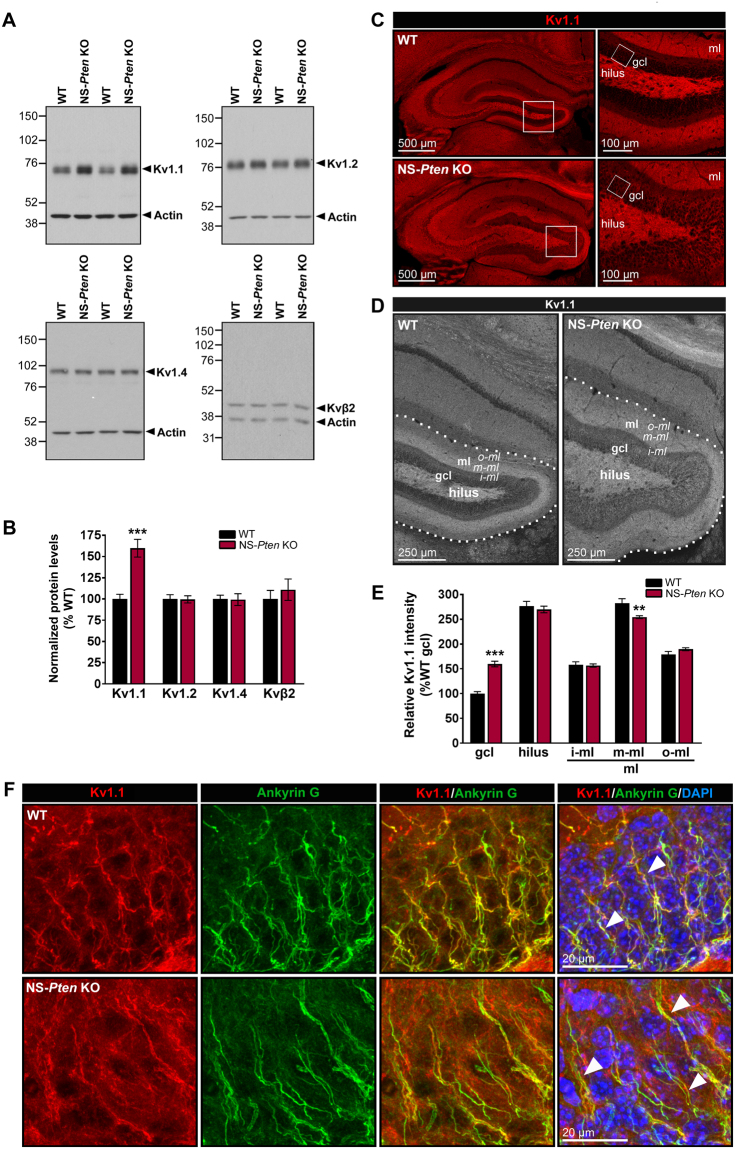
Hippocampal Kv1.1, but not Kv1.2, Kv1.4, or Kvβ2, protein levels are increased in NS-*Pten* KO mice. (**A**) Representative western blots from whole hippocampal homogenates from 6 week-old WT and NS-*Pten* KO mice probed with antibodies against Kv1.1, Kv1.2, Kv1.4, Kvβ2, and actin are shown. (**B**) Quantification of the western blots showed that Kv1.1 protein levels were significantly increased in NS-*Pten* KO compared to WT mice. No changes in Kv1.2, Kv1.4, or Kvβ2 protein levels were observed between NS-*Pten* KO and WT mice. n = 6–8 mice per group; ***p < 0.001 by Student’s t-test; errors bars are ± SEM. (**C**) Representative single optical sections of Kv1.1 immunostaining in 7 week-old WT and NS-*Pten* KO hippocampus are shown. White squares in the left panels outline the areas of the DG that are enlarged in the right panels. White squares in the right panels outline the areas of the DG granule cell layer that are enlarged in panel F. Images represent comparable sections from WT and NS-*Pten* KO mice; note the enlarged NS-*Pten* KO hippocampus, particularly in the DG where loss of Pten is most substantial. (**D**) Representative, grayscale single optical sections of Kv1.1 immunostaining in WT and NS-*Pten* KO mice, enlarged to demonstrate the DG hilus, granule cell layer, and molecular sublayers (inner, middle, outer), are shown. Dotted lines outline the DG. (**E**) Quantification of Kv1.1 immunostaining intensity in the DG show significantly increased Kv1.1 intensity in the granule cell layer and significantly decreased Kv1.1 intensity in the middle molecular layer of NS-*Pten* KO compared to WT mice. No changes in Kv1.1 intensity were observed in the hilus and in the inner and outer molecular layers. n = 7–9 mice per group; **p < 0.01, ***p < 0.001 by Student’s t-test; errors bars are ± SEM. (**F**) High magnification maximum intensity projection images of Kv1.1 and ankyrin G co-immunostaining in WT and NS-*Pten* KO DG granule cell layer are shown. In WT DG granule cell layer, Kv1.1 staining was found to co-localize with ankyrin G staining along the AIS of DG granule cells (arrowheads). Co-localization of Kv1.1 and ankyrin G staining was also found in NS-*Pten* KO DG granule cell layer (arrowheads). In addition, dispersed staining in somatic and perisomatic regions was observed in NS-*Pten* KO DG granule cell layer. Note the presence of cytomegaly and cell dispersion in the DG of NS-*Pten* KO compared to WT mice. n = 6–9 mice per group. *gcl, granule cell layer; ml, molecular layer; i-ml, inner molecular layer; m-ml, middle molecular layer; o-ml, outer molecular layer*.

The Kv1 subfamily of channels consists of multiple members, with Kv1.1, Kv1.2, and Kv1.4 being the most abundant Kv1 α subunits in the mammalian brain^26^. In the hippocampus, precise patterns of Kv1.1, Kv1.2, and Kv1.4 co-localization suggest that these subunits are closely associated with one another as components of heterotetrameric Kv1 channels^17,27^. Kv1 channel complexes are also associated with auxiliary β subunits that help modulate channel function and expression^16^. In rodent brains, Kvβ2 is the most abundantly expressed β subunit^27,28^. Dysregulation of Kv1 α and β subunits has been linked to hyperexcitability and epilepsy^13^; therefore, we evaluated whether the protein expression of Kv1.1, Kv1.2, Kv1.4, and Kvβ2 subunits was altered in hippocampi of NS-*Pten* KO mice. We found that Kv1.1 protein levels were significantly increased in whole hippocampal homogenates from 6-week old NS-*Pten* KO compared to age-matched WT mice by western blotting (p < 0.001 by Student’s t-test). Interestingly, no changes in the protein levels of Kv1.2, Kv1.4, or Kvβ2 were observed between NS-*Pten* KO and WT mice (Fig. 2A,B).

To further evaluate the distribution of Kv1.1 subunits in the hippocampus, we immunostained for Kv1.1 in brain sections from 7 week-old NS-*Pten* KO and WT mice. We narrowed our focus to the DG, given that NS-*Pten* KO mice exhibit deletion of Pten with marked mTOR hyperactivity in DG granule cells (Fig. 1C,D). In the WT DG, intense staining for Kv1.1 was found in the hilus and in the middle third molecular layer, consistent with previously described patterns of normal Kv1.1 distribution^17,26^. Weaker Kv1.1 staining was also found in the inner and outer molecular layers as well as in the granule cell layer. Similarly to the WT DG, NS-*Pten* KO DG also displayed prominent Kv1.1 staining in the hilus and in the middle molecular layer with weaker staining in the inner and outer molecular layers and in the granule cell layer (Fig. 2C,D). Quantification of Kv1.1 staining intensity in the different DG subregions revealed significantly increased Kv1.1 staining in the granule cell layer of NS-*Pten* KO DG compared to WT DG (p < 0.001 by Student’s t-test). Interestingly, we also observed decreased Kv1.1 staining intensity in the middle molecular layer of NS-*Pten* KO DG compared to WT DG (p < 0.01 by Student’s t-test), while no changes were observed in the hilus and in the inner and outer molecular layers between the two groups. (Fig. 2E). High magnification, maximum intensity projection images of the WT DG granule cell layer revealed specific staining for Kv1.1 in string-like structures that co-localized with the AIS marker ankyrin G, indicating that Kv1.1 localizes to the AIS of WT DG granule cells as expected. No staining outside the AIS was observed in the WT DG granule cell layer. Co-localization of Kv1.1 and ankyrin G staining was also found in the NS-*Pten* KO DG granule cell layer. However, additional dispersed staining in somatic and perisomatic regions were observed within the NS-*Pten* KO DG granule cell layer, suggesting the increased Kv1.1 protein levels observed by western blotting may be localized to these regions. (Fig. 2F). Overall, these results demonstrate a specific increase in hippocampal Kv1.1 protein expression, but not other Kv1 subunits, in NS-*Pten* KO mice.

### Aberrant increases in hippocampal Kv1.1 protein levels are present in young adult but not juvenile NS-*Pten* KO mice

**Figure 3:**
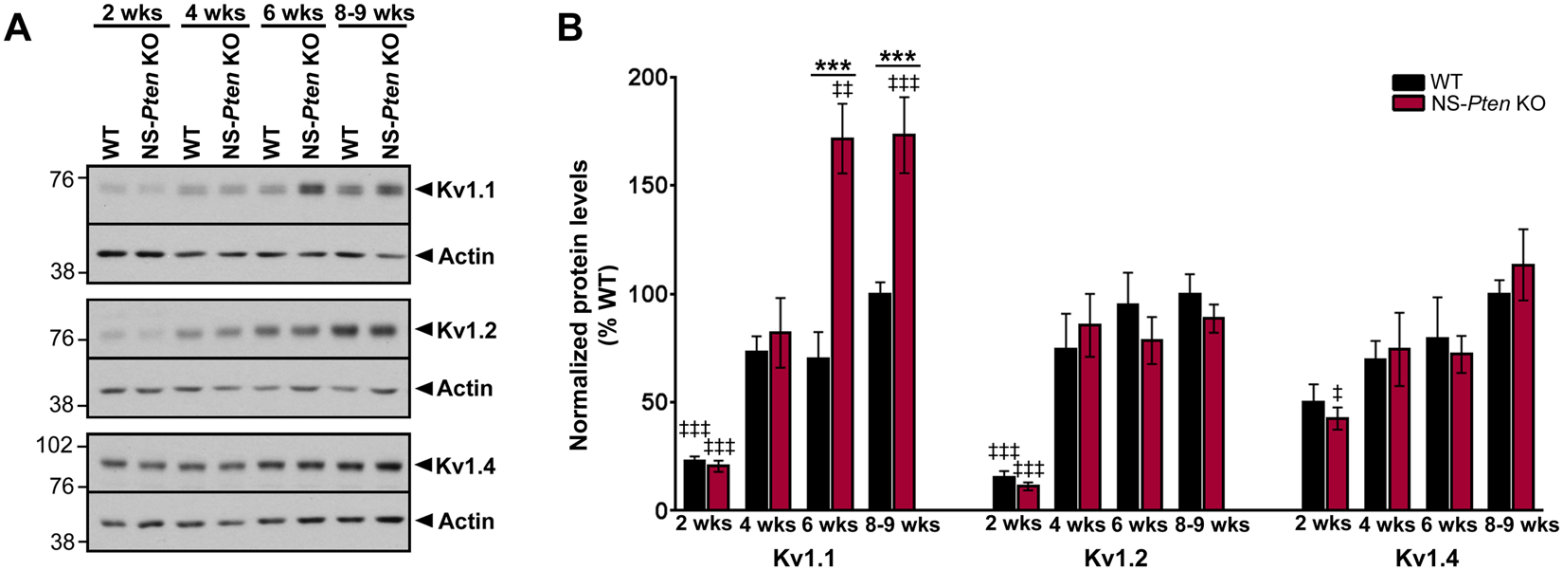
Aberrant increases in hippocampal Kv1.1 protein levels are present in young adult but not juvenile NS-*Pten* KO mice. (**A**) Representative western blots from whole hippocampal homogenates from 2, 4, 6 and 8–9 week-old WT and NS-*Pten* KO mice probed with antibodies against Kv1.1, Kv1.2, Kv1.4, and actin are shown. Full-length blots are presented in Supplemental Fig. 2. (**B**) Quantification of western blots showed that Kv1.1 protein levels were not different between age-matched WT and NS-*Pten* KO and mice at postnatal weeks 2 and 4 (juvenile), but were significantly higher in NS-*Pten* KO compared to WT mice at postnatal weeks 6 and 8–9 (young adults). Additionally, both WT and NS-*Pten* KO mice displayed significantly lower Kv1.1 protein levels at postnatal week 2 compared to adult WT mice at postnatal weeks 8–9. Kv1.2 and Kv1.4 protein levels were not different between age-matched WT and NS-*Pten* KO mice at any time points between postnatal weeks 2 and 8–9. However, both WT and NS-*Pten* KO mice displayed significantly lower Kv1.2 protein levels at postnatal week 2 compared to adult WT mice at postnatal weeks 8–9. n = 6–15 mice per group; ***p < 0.001 (compared to age-matched WT), ^‡^p < 0.05, ^‡‡^p < 0.01, ^‡‡‡^p < 0.001 (compared to 8–9 week-old WT) by two-way ANOVA with Tukey’s post hoc test; errors bars are ± SEM.

NS-*Pten* KO mice exhibit a number of phenotypes, including mTOR hyperactivation, neuronal cytomegaly, cortical and hippocampal disorganization, and epilepsy, which are evident after 4 to 6 weeks of postnatal development and worsen with age^20–22,29^. Therefore, we investigated whether alterations in Kv1.1 protein levels progressed over time in parallel to the previously described phenotypes in NS-*Pten* KO mice. Western blotting was performed on whole hippocampal tissue from WT and NS-*Pten* KO mice collected at postnatal weeks 2, 4, 6, and 8–9. In WT mice, Kv1.1 protein levels were initially low at postnatal week 2 compared to postnatal weeks 8–9, but increased to adult levels by postnatal week 4 (p < 0.001 by two-way ANOVA with Tukey’s post-hoc test). No differences in Kv1.1 protein levels were found between age-matched WT and NS-*Pten* KO mice at postnatal weeks 2 and 4. However, Kv1.1 protein levels were significantly higher in NS-*Pten* KO compared to age-matched WT mice at postnatal weeks 6 and 8–9 (p < 0.001 by two-way ANOVA with Tukey’s post-hoc test) (Fig. 3A,B). Kv1.2 and Kv1.4 protein levels were not different between age-matched WT and NS-*Pten* KO mice at any time points between postnatal weeks 2 and 8–9. However, similar to Kv1.1, both genotypes displayed significantly lower Kv1.2 protein levels at postnatal week 2 compared to adult WT levels at postnatal weeks 8–9 (p < 0.001 by two-way ANOVA with Tukey’s post-hoc test) (Fig. 3A,B). Taken together, these results reveal that aberrant increases in hippocampal Kv1.1 protein levels occur in young adult (≥postnatal week 6) but not juvenile (≤postnatal week 4) NS-*Pten* KO mice, suggesting age-dependent alterations in Kv1.1 protein expression that parallels the previously described progression of mTOR hyperactivation and epileptiform activity in these animals^29^.

### Hippocampal Kv1.1 mRNA (KCNA1) levels are not altered in adult NS-Pten KO mice

**Figure 4:**
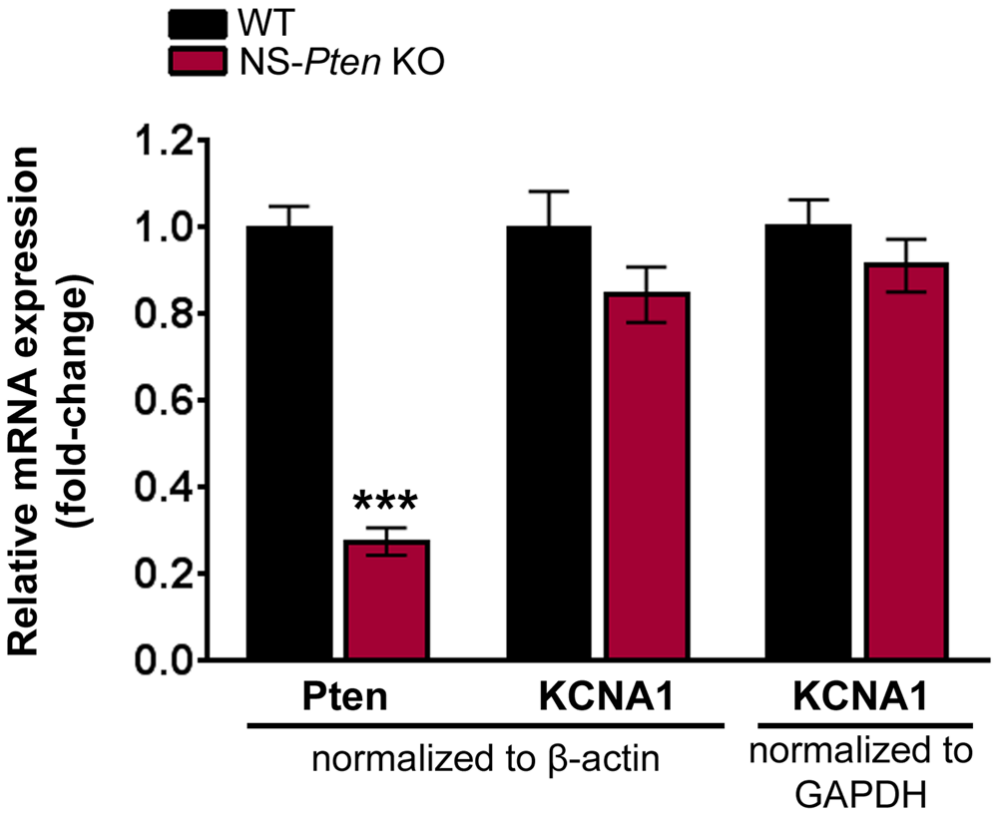
Hippocampal Kv1.1 mRNA (KCNA1) levels are not altered in adult NS-*Pten* KO mice. Relative quantification of Pten and Kv1.1 (KCNA1) mRNA expression using RT-qPCR analysis on total hippocampal RNA isolated from 8 week-old WT and NS-*Pten* KO mice are shown. Pten mRNA levels were significantly decreased in NS-*Pten* KO compared to WT mice. No significant changes in Kv1.1. mRNA levels were observed between NS-*Pten* KO and WT mice. β-actin and GAPDH expression were used as internal controls. n = 7–8 mice per group; ***p < 0.001 by Student’s t-test; errors bars are ± SEM.

Cellular gene expression is intricately regulated by a number of processes spanning multiple levels from mRNA synthesis to protein post-translational modification and localization^30^. In addition, the maintenance of protein expression is also regulated by protein degradation pathways^31^. Presumably, the observed increase in Kv1.1 protein levels in NS-*Pten* KO mice can result from dysregulation of any of these regulatory processes or an imbalance between synthesis and degradation mechanisms (altered turnover) that cause more proteins to be made than removed. As an initial insight into whether increased Kv1.1 protein expression in NS-*Pten* KO mice reflects dysregulation at the level of mRNA or protein, we investigated whether hippocampal Kv1.1 mRNA levels were altered in adult NS-*Pten* KO mice. Total RNA was isolated from whole hippocampal tissue from 8 week-old WT and NS-*Pten* KO mice and real-time quantitative PCR analysis was performed to quantify the levels of Kv1.1 mRNA relative to two internal control genes, β-actin and GAPDH, within each sample. As a positive control, we first confirmed that Pten mRNA levels were decreased in NS-*Pten* KO mice. As expected, Pten mRNA levels were significantly reduced in NS-*Pten* KO compared to WT mice (p < 0.001 by Student’s t-test). However, we found no significant changes in Kv1.1 mRNA (KCNA1) levels between NS-*Pten* KO and WT mice (Fig. 4). These results indicate that Kv1.1 alterations do not occur at the level of mRNA synthesis, and the increase in Kv1.1 protein expression likely involves mechanisms that occur after Kv1.1 mRNAs are generated, possibly due to aberrant regulation of protein synthesis or degradation.

### Rapamycin treatment normalizes Kv1.1 protein levels in NS-*Pten* KO mice

To determine whether increased Kv1.1 protein levels are associated with hyperactive mTOR signaling in NS-*Pten* KO mice, we evaluated whether mTOR suppression with a pharmacological inhibitor, rapamycin, reduces Kv1.1 protein levels in these mice. WT and NS-*Pten* KO mice were treated with rapamycin according to a paradigm that was previously shown to suppress mTORC1 and mTORC2 signaling in WT and NS-*Pten* KO brains (10 mg/kg i.p. injections per day, 5 days per week for 2 weeks)^29^. A cohort of animals was treated early, during postnatal weeks 4 and 5, while another cohort of animals was treated late, during postnatal weeks 9 and 10. In the first experiment, early rapamycin treatment was initiated at an age before Kv1.1 alterations were present to test if the pathology could be prevented. In the second experiment, late rapamycin treatment was initiated after Kv1.1 alterations were already in place to investigate if the pathology could be reversed once it had occurred. Whole hippocampal tissue was collected for western blot analysis one day after the last rapamycin injection, at postnatal week 6 and 11 for the early and late treatment experiments, respectively.

**Figure 5:**
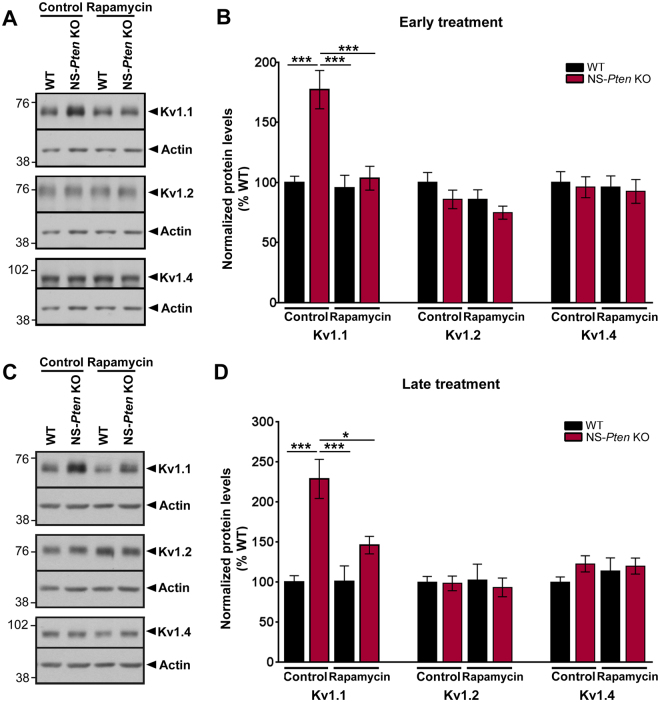
Rapamycin treatment normalizes Kv1.1 protein levels in NS-*Pten* KO mice. WT and NS-*Pten* KO mice were treated with vehicle or rapamycin (10 mg/kg i.p.) for 5 days per week for 2 weeks, either during postnatal weeks 4 and 5 (**A,B**; early treatment) or during postnatal weeks 9 and 10 (**C,D**; late treatment). Tissue was collected for western blotting one day after the last treatment. (**A**) Representative western blots from whole hippocampal homogenates from early control (naïve and vehicle-treated) and rapamycin-treated WT and NS-*Pten* KO mice probed with antibodies against Kv1.1, Kv1.2, Kv1.4, and actin are shown. Full-length blots are presented in Supplemental Fig. 2. (**B**) Quantification of western blots showed that Kv1.1 protein levels were significantly higher in NS-*Pten* KO compared to WT mice in the control group. Early rapamycin treatment suppressed Kv1.1 protein levels in NS-*Pten* KO mice to WT levels. No significant changes in Kv1.2 or Kv1.4 protein levels were found between NS-*Pten* KO and WT mice regardless of treatment group. n = 7–16 mice/group; ***p < 0.001 by one-way ANOVA with Tukey’s post-hoc test; errors bars are ± SEM. (**C**) Representative western blots from whole hippocampal homogenates from late control (naïve and vehicle-treated) and rapamycin-treated WT and NS-*Pten* KO mice probed with antibodies against Kv1.1, Kv1.2, Kv1.4, and actin are shown. Full-length blots are presented in Supplemental Fig. 2. (**D**) Quantification of western blots showed that Kv1.1 protein levels were significantly higher in NS-*Pten* KO compared to WT mice in the control group. Late rapamycin treatment suppressed Kv1.1 protein levels in NS-*Pten* KO mice to WT levels. No significant changes in Kv1.2 or Kv1.4 protein levels were found between NS-*Pten* KO and WT mice regardless of treatment group. n = 6–14 mice/group; *p < 0.05, ***p < 0.001 by one-way ANOVA with Tukey’s post-hoc test; errors bars are ± SEM.

Naïve and vehicle-treated control NS-*Pten* KO mice displayed significantly increased Kv1.1 protein levels compared to age-matched control WT mice in both experiments (p < 0.001 by one-way ANOVA with Tukey’s post-hoc test) (Fig. 5A–D). NS-*Pten* KO mice that received rapamycin treatment during postnatal weeks 4 and 5 displayed significantly lower Kv1.1 protein levels compared to age-matched control NS-*Pten* KO mice by postnatal week 6 (p < 0.001 by one-way ANOVA with Tukey’s post-hoc test). Moreover, Kv1.1 protein levels in the early rapamycin-treated NS-*Pten* KO mice were similar to that of age-matched WT control mice, indicating that early rapamycin treatment in juvenile NS-*Pten* KO mice prevented the aberrant increase in Kv1.1 protein levels from occurring (Fig. 5A,B). NS-*Pten* KO mice that received rapamycin treatment during postnatal weeks 9 and 10 also displayed significantly lower Kv1.1 protein levels compared to age-matched control NS-*Pten* KO mice by postnatal week 11 (p < 0.05 by one-way ANOVA with Tukey’s post-hoc test). Similarly to early treatment, no differences in Kv1.1 protein levels were observed between the late rapamycin-treated NS-*Pten* KO mice and age-matched control WT mice, indicating that late rapamycin treatment in adult NS-*Pten* KO mice reversed the ongoing increases in Kv1.1 protein levels to WT levels (Fig. 5C,D). Rapamycin treatment had no effects on Kv1.1 protein levels in WT mice in both experiments. In addition, no significant changes in Kv1.2 or Kv1.4 protein levels were found in NS-*Pten* KO and WT mice regardless of treatment group (Fig. 5A–D). Taken together, these results demonstrate that rapamycin treatment both before and after the onset of Kv1.1 abnormalities normalizes aberrant Kv1.1 protein levels in NS-*Pten* KO mice and suggest that altered Kv1.1 protein expression is modulated by mTOR activity in this model.

## Discussion

Despite extensive research demonstrating a role for the mTOR pathway in CD and other MCDs, the underlying mechanisms by which mTOR hyperactivation lead to epilepsy in these disorders remain unclear. Epilepsy is characterized by recurrent seizures presumably arising from rewiring of the brain circuitry into hyperexcitable networks^32^. At the cellular level, hyperexcitability may originate from dysregulation of voltage-gated ion channels which are critical regulators of neuronal excitability^33^. Given the emerging involvement for mTOR signaling in voltage-gated ion channel expression^14,34^, one candidate mechanism by which mTOR dysregulation promotes epilepsy in CD is through remodeling of voltage-gated ion channel expression. Here, we evaluated whether constitutive mTOR hyperactivation is associated with altered voltage-gated ion channel expression in the NS-*Pten* KO mouse model of CD. We found that hippocampal Kv1.1, but not Kv1.2, Kv1.4, or Kvβ2, protein expression was increased in NS-*Pten* KO mice. The changes in Kv1.1 expression were observed in young adult but not juvenile NS-*Pten* KO mice, and were specific to proteins as no changes in mRNA levels were found. Furthermore, early and late mTOR inhibition with rapamycin treatment normalized Kv1.1 protein levels in NS-*Pten* KO mice. Collectively, these studies demonstrate increased Kv1.1 protein expression in association with mTOR hyperactivation and support a role for mTOR signaling in the modulation of Kv1 channel expression in the NS-*Pten* KO mouse model.

The previously described precise patterns of Kv1.1, Kv1.2, Kv1.4, and Kvβ2 co-localization in rodent hippocampus suggest a close interaction between these subunits, likely as components of heterotetrameric channels^17,27^. Kv1 subunits are primarily localized in axons; however, they have also been found in somatodendritic regions of some neurons^35^. Given that the observed increases in protein expression were specific to the Kv1.1 subunit in NS-*Pten* KO mice, the mechanisms for increased Kv1.1 are likely not due to global structural changes associated with Pten loss, such as increased soma, axon, or dendrite size^36^, but instead due to a regulatory mechanism that is specific to this subunit. In mammalian cells, the production and maintenance of proteins are regulated by multiple processes, including DNA-to-RNA transcription, mRNA processing and translation, and post-translational protein modification, localization, and degradation^30,31^. Our data reveal increased Kv1.1 protein but not mRNA levels, thus supporting a mechanism involved after mRNA generation, such as increased mRNA translation and protein synthesis, decreased protein degradation, or imbalanced protein turnover. mTOR signaling mediates protein synthesis through mRNA translational control and could potentially regulate Kv1.1 expression through these mechanisms^5^. Interestingly, a previous study showed that mTOR activity mediates local repression of dendritic Kv1.1 mRNA translation in rat hippocampus by microRNA miR-129–5p binding. When mTOR activity was reduced, miR-129-5p binding was relieved and dendritic Kv1.1 mRNA translation was promoted^14,34^. In contrast, our results showed an overall increase in hippocampal Kv1.1 proteins level in association with increased mTOR signaling. These dissimilar results may reflect a difference in dendritic versus axonal mechanisms since mTOR suppression with rapamycin increased dendritic but not axonal Kv1.1 expression in rat hippocampus^14^. These observations may also reflect differences in mTOR functions under physiological versus pathological conditions or species differences. Future studies to elucidate the molecular mechanisms by which mTOR hyperactivity contribute to increased Kv1.1 protein expression in NS-*Pten* KO mice will be important. Furthermore, since the rapamycin treatment paradigm used in our studies suppressed both mTORC1 and mTORC2 signaling, it is unclear whether the observed effects on Kv1.1 were due to suppression of mTORC1, mTORC2, or both, and additional studies will also be needed to dissect the roles of mTORC1 versus mTORC2.

Voltage-gated potassium channels function to hyperpolarize neurons, and increased voltage-gated potassium channel expression in glutamatergic neurons, including DG granule cells, is predicted to decrease network excitation^37^. Therefore, it may appear counterintuitive that NS-*Pten* KO mice with epilepsy exhibit increased Kv1.1 protein expression. However, an increase in Kv1.1 subunits may not necessarily reflect increased Kv1 channel function, as studies have shown that the subunit composition and stoichiometry of Kv1 channel complexes considerably influences their expression and function. For example, in cultured hippocampal neurons, Kv1 channels consisting of only Kv1.1 subunits (Kv1.1 homotetramers) do not get trafficked to the cell surface, where channels normally function, whereas Kv1 channels consisting of Kv1.1 and Kv1.4 subunits (Kv1.1:Kv1.4 heterotetramers) do traffic to the cell surface, where they are expressed and functional^38^. Consistent with this idea, we observed increased Kv1.1 staining in somatic and perisomatic regions in DG granule cells from NS-*Pten* KO but not WT mice, suggesting aberrant Kv1.1 localization in NS-*Pten* KO mice. Furthermore, an imbalance in the pool of existing Kv1 subunits, caused by an increase in Kv1.1 but not the other Kv1 subunits, may disrupt the subunit stoichiometry and potentially the function of Kv1 channels containing these subunits. Based on the above, we speculate that, despite an increase in Kv1.1 subunits, there is reduced Kv1 function in NS-*Pten* KO hippocampus. Interestingly, hippocampal neurons lacking Pten exhibit increased action potential duration *in vitro*^39,40^, a finding that is consistent with loss of Kv1 function^41–43^. In the present study, direct electrophysiological assessment of Kv1.1 function in Pten-negative neurons with hyperactive mTOR signaling was not performed. Therefore, the functional link between altered Kv1.1 expression and hyperexcitability in this model remains undefined and is a direction for future studies.

The DG is thought to function as a gate controlling the flow of excitatory inputs from the entorhinal cortex into the hippocampus, and dysfunction of the DG gate has been hypothesized to promote epilepsy^44–46^. Potentially, Kv1 dysregulation may contribute to disrupted DG gating functions. Previously, it has been shown that the same rapamycin treatment paradigms used in the present study effectively suppressed epilepsy in NS-*Pten* KO mice^20,29,47^. Thus, our findings pose an interesting question for future investigation as to whether some of the effects of rapamycin on epileptiform activity are due to Kv1 ion channel modulation. Interestingly, a previous study has reported increased expression of small conductance calcium-activated potassium channels (SK_Ca_) resulting in decreased intrinsic excitability and firing rate in cortical neurons lacking one copy of Pten, which further supports a role for Pten and the mTOR pathway in the modulation of neuronal ion channel expression and function^48^. Building upon our results and those of others, future electrophysiological experiments to determine functional consequences of altered hippocampal Kv1.1 protein expression and the contribution to seizure circuitry in NS-*Pten* KO mice will be important.

Alterations in ion channels have been described following seizures^49^. Thus, it is possible that increased Kv1.1. protein expression reflects an activity-dependent, secondary effect of seizures as increased Kv1.1 protein levels were found at an age where epileptiform activity was also evident^29^. Normalization of altered Kv1.1 protein expression by rapamycin treatment may therefore be an indirect effect of reduced seizure frequency, as rapamycin treatment has been shown to suppress seizures in NS-*Pten* KO mice^20,29,47^. However, the specific effect of seizures on Kv1.1 expression is unclear. In the rat kainate model of status epilepticus (SE), increased Kv1.1 protein levels were observed 2 weeks after SE while decreased Kv1.1 protein levels were found 30 days after SE^50^. Increased Kv1.1 protein expression was also found in mice 20 days after intrahippocampal injections with kainate, a finding thought to be an anti-epileptic, homeostatic mechanism^51^. In contrast, previous work from our lab revealed no changes in Kv1.1 protein levels 2 weeks after SE in the rat pilocarpine model of SE^52^. Furthermore, Kv1.1 mRNA levels were unchanged in rats following seizure induction by pentylenetetrazole^53^. Thus, future studies aimed to separate the consequences of mTOR dysregulation versus seizures in NS-*Pten* KO mice are undeniably important to understand the extent by which Kv1.1 expression is controlled by mTOR activity and recurrent seizures in this model. Blocking seizures with a conventional anti-epileptic drug (AED) that does not act on the mTOR pathway seems to be a straightforward approach to separate the effects of mTOR dysregulation from seizures. However, finding a drug that suppresses seizures in NS-*Pten* KO mice has been challenging as these animals seem to be resistant to conventional AEDs, such as phenobarbital, much like humans with intractable epilepsy associated with MCDs (unpublished observations). Alternative genetic approaches where Pten is sparsely deleted in a smaller number of neurons, so as not to cause a widespread epilepsy phenotype, may be more advantageous to separate the effects of mTOR dysregulation and seizures.

In conclusion, our studies provide biochemical and histological evidence for altered Kv1.1 protein expression in association with mTOR hyperactivation in the NS-*Pten* KO mouse model of CD, and we hypothesize that mTOR-dependent ion channel dysregulation is a potential contributor to CD-related epilepsy. A previous study identifying increased expression and mislocalization of Kv1.1 in cell bodies with reduced axonal expression in brain tissue from an individual with CD and intractable epilepsy support the potential clinical relevance of our studies^54^. Nonetheless, future electrophysiological studies will be important to identify the functional relevance of altered Kv1.1 protein expression to epilepsy in the present model.

## Materials and Methods

### Animals

Neuron subset-specific *Pten* conditional knockout (NS-*Pten* KO) mice have been described previously as *Pten*^loxP/loxP^; Gfap-Cre mice^20–22^ and are on a FVB‐based backcrossed background strain that had been bred for more than 10 generations (RRID:MGI:3714016). Littermate NS-*Pten* KO (*Pten*^+/+^; Gfap-Cre) and wildtype (WT; *Pten*^+/+^; Gfap-Cre) mice were generated by breeding heterozygote (*Pten*^loxP/−^; Gfap-Cre) mice. Both male and female mice were used for all experiments. The specific animal number and sex for each experiment are listed in Supplemental Table 1. Mice were deeply anesthetized with a ketamine/xylazine/acepromazine combination prior to tissue collection for all experiments. Animal care and use were in compliance with the National Institutes of Health *Guidelines for the Care and Use of Laboratory Animals*. All experimental protocols were approved by the Institutional Animal Care and Use Committee at Baylor College of Medicine and performed in accordance with the approved guidelines.

### Western blotting

Whole hippocampi were rapidly dissected in ice-cold phosphate buffered saline (PBS; 137 mM NaCl, 2.7 mM KCl, 4.3 mM Na_2_HPO_4_, 1.47 mM KH_2_PO_4_, pH 7.4) and flash frozen in dry ice. All samples were stored at −80 °C until used. Hippocampi were homogenized in ice-cold homogenization buffer (100 mM Tris-HCl, pH 7.4, 0.32 M sucrose, 1 mM EDTA, 5 mM HEPES) containing protease and phosphatase inhibitor cocktail tablets (Roche). Total protein concentration of the samples was determined using Bradford protein assay (Bio-Rad Laboratories). Samples were prepared for SDS-PAGE by dilution in Laemmli buffer (4×: 0.25 M Tris-HCl, pH 6.8, 6% SDS, 40% sucrose, 0.04% bromophenol blue, 200 mM dithiothreitol) and 100 mM Tris-HCl, pH 7.4. Equal amount of proteins (10 µg) per sample were resolved by SDS-PAGE and transferred onto PVDF membranes (GE Healthcare). Membranes were incubated in blocking buffer (5% non-fat milk, 1 mM Na_3_VO_4_ in PBS + 0.1% Tween 20) for 1 hour at room temperature, in primary antibodies overnight at 4 °C, and in secondary antibodies for 1 hour at room temperature. Membranes were washed 3 × 10 min in PBS + 0.1% Tween 20 between each step. Immunoreactive bands were developed using enhanced chemiluminescence reagents (Thermo Scientific) and captured on autoradiography films (Phenix Research Products). Optical densities of immunoreactive bands were measured using Image J software (National Institutes of Health). Each band was normalized to the actin band within the same lane for loading control. Phospho-proteins were subsequently normalized to the respective total protein level. Results were expressed as percentages of age-matched WT mice (Figs 1B and 2B), 8–9 week-old adult WT mice (Fig. 3B), or control WT mice (Fig. 5B,D).

### Immunostaining and image analysis

For Pten and p-S6 (S240) immunostaining, mice were perfused with 4% paraformaldehyde (PFA). Brains were post-fixed in 4% PFA overnight, cryoprotected in 30% sucrose until brains sank, and then frozen in pre-chilled isopentane on dry-ice. Free-floating 40 μm-thick coronal sections were washed 2 × 10 min in PBS + 0.1% triton X-100 and permeabilized for 20 min in PBS + 1% triton X-100. Sections were incubated in blocking buffer (5% goat serum, 0.3% BSA, 0.3% triton X-100 in PBS) for 2 hours at room temperature and incubated in primary antibodies for 3 nights at 4 °C. Sections were then washed 3 × 10 min in PBS + 0.1% triton X-100 and incubated in secondary antibodies for 1.5 hours, followed by incubation in DAPI for 10 min to stain for cell nuclei. Sections were put through a final 3 × 30 min wash in PBS + 0.1% triton X-100, mounted onto slides, air-dried, and coverslipped with fluorescent mounting medium (Dako). Staining was imaged using a Zeiss LSM 710 confocal system. For quantification of Pten and p-S6 (S240) staining intensity, 6 representative regions of interest (ROIs) of equal area were selected along the DG granule cell layer from 1 brain section and the mean gray values of the ROIs were measured using Image J. Results were expressed as percentages of WT intensity (Fig. 1D).

For Kv1.1 immunostaining, whole brains were rapidly dissected in ice-cold PBS and drop-fixed in 4% PFA for 2 hours. Fixed brains were cryoprotected in 30% sucrose until they sank and rapidly frozen in OCT compound using a dry ice-ethanol bath. 30 μm-thick coronal brain sections mounted on slides were washed 2 × 10 min in PBS + 0.1% triton X-100, incubated 5 min in deionized H_2_0, and treated with pepsin (0.2 mg/ml in 0.2 M HCl; Dako) for 10 min at 37 °C for antigen retrieval^55^. Sections were then washed 3 × 10 min in PBS + 0.1% triton X-100 and incubated in blocking buffer (10% goat serum, 0.3% BSA, 0.3% triton X-100 in PBS) for 2 hours. Sections were incubated in primary antibodies overnight, followed by 3 × 10 min washes in PBS + 0.1% triton X-100, and incubation in secondary antibodies for 1 hour. Sections were incubated in DAPI for 10 min, washed 3 × 10 min and 3 × 30 min in PBS + 0.1% triton X-100, and rinsed in PBS before being air-dried and coverslipped with fluorescent mounting medium (Dako). All incubation and washing steps were performed at room temperature. Staining was imaged using a Zeiss LSM 880 confocal system with airyscan. Low magnification single optical sections were acquired from 3–6 brain sections per animal from a total of 7 WT and 9 NS-*Pten* KO mice. For quantification of Kv1.1 staining intensity, 3 brain sections per animal were analyzed. For each brain section, 3 representative ROIs of equal area were selected along each of the DG hilus, granule cell layer and molecular sublayers (inner, middle, outer) and the mean gray values of the ROIs were measured using Image J. Results were expressed as percentages of WT gcl intensity (Fig. 2E). High magnification z-stacks were acquired from 3 different areas along the DG granule cell layer per brain section, 2 brain sections per animal from a total of 6 WT and 9 NS-*Pten* KO mice. Maximum intensity projection images were created from a 10 µm-thick z-stack of optical sections taken at 0.5 µm increments using Zeiss ZEN microscope software built-in tools. All images directly compared were uniformly processed.

### Antibodies

The following primary antibodies were used for western blotting: rabbit anti-Pten (1:2000, Cell Signaling #9188), rabbit anti-p-S6 (S240/244) (1:2000, Cell Signaling #5364), rabbit anti-S6 (1:2000, Cell Signaling #2217), rabbit anti-p-AKT (S473) (1:2000, Cell Signaling #4060), rabbit anti-p-AKT (T308) (1:2000, Cell Signaling #2965), rabbit anti-AKT (1:2000, Cell Signaling #9272), mouse anti-Kv1.1 (1:1000, Neuromab #75-007, clone K20/78), mouse anti-Kv1.2 (1:1000, Neuromab #75-008, clone K14/16), mouse anti-Kv1.4 (1:1000, Neuromab #75-010, clone K13/31), mouse anti-Kvβ2 (1:1000, Neuromab #75-021, clone K17/70), and rabbit anti-actin (1:5000, Sigma-Aldrich #A2066). The following secondary antibodies were used for western blotting: horseradish peroxidase-linked goat anti-rabbit IgG (1:2000, Cell Signaling #7074) and anti-mouse IgG (1:2000, Cell Signaling #7076).

The following primary antibodies were used for immunostaining: mouse anti-p-S6 (S240) (1:100, Dako #M7300, clone DAK-S6-240), rabbit anti-Pten (1:100, Cell Signaling #9188), mouse anti-Kv1.1 (1:200, Neuromab #75-105, clone K36/15, external), and mouse anti-ankyrin G (1:200, Neuromab #75-146, clone N106/36). The following secondary antibodies were used for immunostaining: Alexa Fluor-conjugated goat anti-rabbit IgG (594) (1:500, Invitrogen #A11037), anti-mouse IgG (488) (1:500, Invitrogen #A11029), anti-mouse IgG2b (594) (1:500, Invitrogen #A21145), and anti-mouse IgG2a (488) (1:500, Invitrogen #A21131). All antibodies were diluted in the respective blocking buffer for western blotting and immunostaining. The specificity of the Kv1.1 antibodies was verified by the absence of immunoreactivity in Kv1.1 KO mice (Supplemental Fig. 1).

### Real-time quantitative PCR (qPCR)

Total RNA was extracted from whole hippocampal tissue using the miRNeasy Mini Kit (Qiagen). RNA quantity and quality were assessed using a NanoDrop 2000c spectrophotometer (Thermo Scientific). RNA concentrations ranged from 147.6 to 250.1 ng/µl for WT samples and 188.6 to 538.8 ng/µl for NS-*Pten* KO samples. A260/280 ratios ranged from 1.99 to 2.09. RNA integrity was also evaluated using agarose gel electrophoresis; all samples displayed sharp 28S and 18S rRNA bands. RNA samples were treated with DNase I (Invitrogen) to remove any contaminating genomic DNA and converted to cDNA by reverse transcription using the SuperScript VILO cDNA Synthesis Kit (Invitrogen). qPCR was performed using the Taqman Fast Advanced Master Mix (Applied Biosystems) and a CFX96 real-time PCR detection system (Bio-Rad Laboratories). cDNA equivalent to 25 ng of input RNA was added per reaction. Primers (Taqman gene expression assays; Applied Biosystems) for qPCR were as follows: Pten (Mm00477208_m1), KCNA1 (Mm00439977_s1), β-actin (Mm00607939_s1), and GAPDH (Mm99999915_g1). All experiments were performed according to the manufacturer’s protocol. Samples were repeated in triplicates. Controls with no reverse transcriptase and no template were included for each gene and each experiment. Raw C_T_ (threshold cycle) values for all amplified genes were within 20 to 29 cycles. Fold-changes in gene expression were calculated using the comparative C_T_ method, where fold-change = 2^−ΔΔC^_T_ = 2^−[(C^_T_^(gene of interest, WT) −C^_T_^(internal control, WT)]−[(C^_T_^(gene of interest, KO)−C^_t_^(internal control, KO)]^ [gene of interest = Pten or KCNA1, internal control = β-actin or GAPDH]^56^.

### Rapamycin treatment

Rapamycin (LC Laboratories) was dissolved in a vehicle solution of 4% ethanol, 5% polyethylene glycol 400, and 5% Tween 80^20^. WT and NS-*Pten* KO mice were treated with rapamycin (10 mg/kg body weight) or vehicle by intraperitoneal (i.p.) injections 5 days per week for 2 weeks, either during postnatal weeks 4 and 5 (early treatment) or during postnatal weeks 9 and 10 (late treatment). Tissue was collected for western blotting the day after the last injection. For each of the treatment studies, no statistical differences were observed between naïve and vehicle-treated mice within each genotype (early treatment: p = 0.9601 for WT, p = 0.9996 for NS-*Pten* KO; late treatment: p = 0.1485 for WT, p = 0.2119 for NS-*Pten* KO); therefore, naïve and vehicle-treatment mice were combined into one control group for each genotype. Each of the WT and NS-*Pten* KO control groups include 14–16 mice consisting of 7–8 vehicle-treated and 6–8 naïve mice.

### Statistics

Statistical analyses were performed using Prism 6 software (GraphPad). Specific tests (Student’s t-test, one-way ANOVA, or two-way ANOVA with Tukey’s post hoc test) were used as noted in the figure legends. The significance level was set at p < 0.05. Data are presented as mean ± standard error of the mean (SEM).

## Electronic supplementary material

Supplementary Information
