# Supplementary material for: mTOR-dependent alterations of Kv1.1 subunit expression in the neuronal subset-specific Pten knockout mouse model of cortical dysplasia with epilepsy
Source: Sci Rep. 2018 Feb 23;8:3568. doi: 10.1038/s41598-018-21656-8 (PMC5824782; doi:10.1038/s41598-018-21656-8)
Supplement: Supplementary file 1 — Supplementary Information [file 41598_2018_21656_MOESM1_ESM.pdf]

SUPPLEMENTARY INFORMATION

mTOR-dependent alterations of Kv1.1 subunit expression in the neuronal subset-specific *Pten* knockout mouse model of cortical dysplasia with epilepsy

Lena H. Nguyen and Anne E. Anderson*

Supplemental Table 1: Animal number and sex for each experiment

**Experiment**	**Animal number and sex**
Figure 1 A, B:	8 WT (4 males, 4 females), 8 NS-*Pten* KO (1 male, 6 females)
Figure 1 C-D:	3 WT, 3 NS-*Pten* KO (sex unknown)
Figure 2 A, B:	8 WT (4 males, 4 females) and 7 NS-*Pten* KO (4 males, 2 females, 1 unknown) ***for Kv1.1, Kv1.2, Kv1.4***<br><br>7 WT (4 males, 3 females) and 6 NS-*Pten* KO (3 males, 2 females, 1 unknown) ***for KvB2***
Figure 2 C-E:	7 WT (4 males, 3 females) and 9 NS-*Pten* KO (6 males, 3 females)
Figure 2 F:	6 WT (4 males, 2 females) and 9 NS-*Pten* KO (6 males, 3 females)
Figure 3A, B:	7 WT-2 wks (unknown sex; pre-weaning age), 7 NS-*Pten* KO-2 wks (unknown sex; pre-weaning age), 7 WT-4 wks (4 males, 3 females), 7 NS-*Pten* KO-4 wks (3 males, 4 females), 6 WT-6 wks (4 males, 2 females), 7 NS-*Pten* KO-6 wks (4 males, 2 females, 1 unknown), 15 WT-8-9 wks (10 males, 5 females), 12 NS-*Pten* KO-8-9 wks (7 males, 3 females, 2 unknown) ***for Kv1.1, Kv1.4***<br><br>7 WT-2 wks (unknown sex; pre-weaning age), 7 NS-*Pten* KO-2 wks (unknown sex; pre-weaning age), 7 WT-4 wks (4 males, 3 females), 7 NS-*Pten* KO-4 wks (3 males, 4 females), 6 WT-6 wks (4 males, 2 females), 6 NS-*Pten* KO-6 wks (4 males, 2 females), 15 WT-8-9 wks (10 males, 5 females), 12 NS-*Pten* KO-8-9 wks (7 males, 3 females, 2 unknown) ***for Kv1.2***
Figure 4:	8 WT (5 males, 3 females) and 7 NS-*Pten* KO (5 males, 2 females, 1 unknown)
Figure 5 A, B:	15 WT-control (8 naïve, 7 vehicle; 8 males, 7 females), 15 NS-*Pten* KO-control (8 naïve, 7 vehicle; 11 males, 3 females, 1 unknown), 8 WT-rapamycin (3 males, 4 females, 1 unknown), 8 NS-*Pten* KO-rapamycin (5 males, 3 females) ***for Kv1.1***<br><br>16 WT-control (8 naïve, 8 vehicle; 9 males, 7 females), 14 NS-*Pten* KO-control (7 naïve, 7 vehicle; 11 males, 3 females), 8 WT-rapamycin (3 males, 4 females, 1 unknown), 8 NS-*Pten* KO-rapamycin (5 males, 3 females) ***for Kv1.2***<br><br>16 WT-control (8 naïve, 8 vehicle; 9 males, 7 females), 15 NS-*Pten* KO-control (8 naïve, 7 vehicle; 11 males, 3 females, 1 unknown), 7 WT-rapamycin (3 males, 4 females), 8 NS-*Pten* KO-rapamycin (5 males, 3 females) ***for Kv1.4***
Figure 5 C, D:	14 WT-control (7 naïve, 7 vehicle; 5 males, 7 females, 2 unknown), 14 NS-*Pten* KO-control (6 naïve, 8 vehicle; 8 males, 4 females, 2 unknown), 7 WT-rapamycin (5 males, 2 females), 7 NS-*Pten* KO-rapamycin (4 males, 3 females) ***for Kv1.1***<br><br>14 WT-control (7 naïve, 7 vehicle; 5 males, 7 females, 2 unknown), 14 NS-*Pten* KO-control (6 naïve, 8 vehicle; 8 males, 4 females, 2 unknown),), 6 WT-rapamycin (4 males, 3 females), 7 NS-*Pten* KO-rapamycin (4 males, 3 females) ***for Kv1.2***<br><br>13 WT-control (6 naïve, 7 vehicle; 5 males, 6 females, 2 unknown), 14 NS-*Pten* KO-control (6 naïve, 8 vehicle; 8 males, 4 females, 2 unknown), 7 WT-rapamycin (5 males, 2 females), 7 NS-*Pten* KO-rapamycin (4 males, 3 females) ***for Kv1.4***

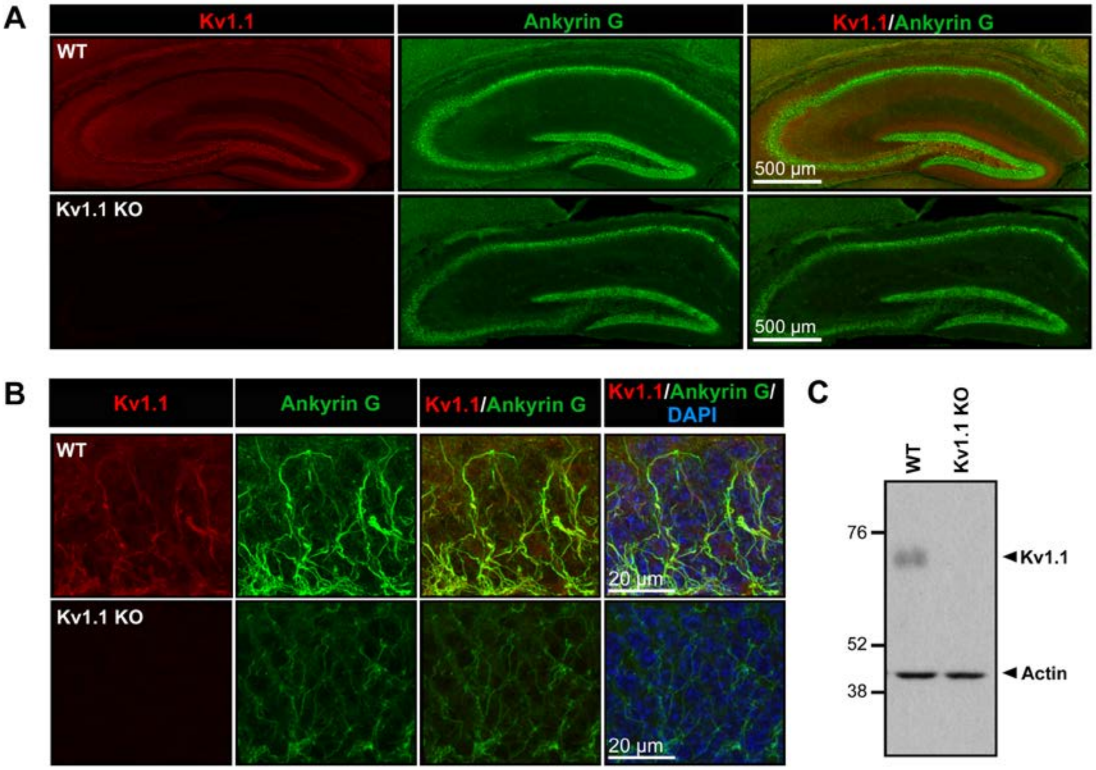

Supplemental figure 1: Validation of Kv1.1 antibody specificity. The specificity of Kv1.1 antibodies was validated using WT (Kv1.1$^{+/+}$; Tau$^{+/-}$) and Kv1.1 KO (Kv1.1$^{-/-}$; Tau$^{+/-}$) mice (generously provided to us by Dr. Jeffrey Noebels at Baylor College of Medicine). **(A)** Single confocal optical sections from WT and Kv1.1 KO hippocampus immunstained with Kv1.1 (1:200, clone K36/15, NeuroMab) and ankyrin G (1:200, clone N106/34, NeuroMab) are shown. Prominent Kv1.1 staining was found in WT hippocampus while no Kv1.1 staining was observed in Kv1.1 KO hippocampus. Ankyrin G staining was present in both WT and Kv1.1 KO hippocampi. **(B)** High magnification maximum intensity projection images of Kv1.1 and ankyrin G co-immunostaining in WT and Kv1.1 KO DG granule cell layer are shown. In WT DG granule cell layer, Kv1.1 staining was found to co-localize with ankyrin G staining. In contrast, no Kv1.1 staining was detected in Kv1.1 KO DG granule cell layer. **(C)** Western blots from whole hippocampal homogenates from WT and Kv1.1 KO mice probed with antibodies against Kv1.1 (1:1000, clone K20/79, NeuroMab) and actin (1:5000, Sigma-Aldrich, loading control) are shown. Kv1.1 immunoreactivity was detected in hippocampal homogenates from WT but not Kv1.1 KO mice.

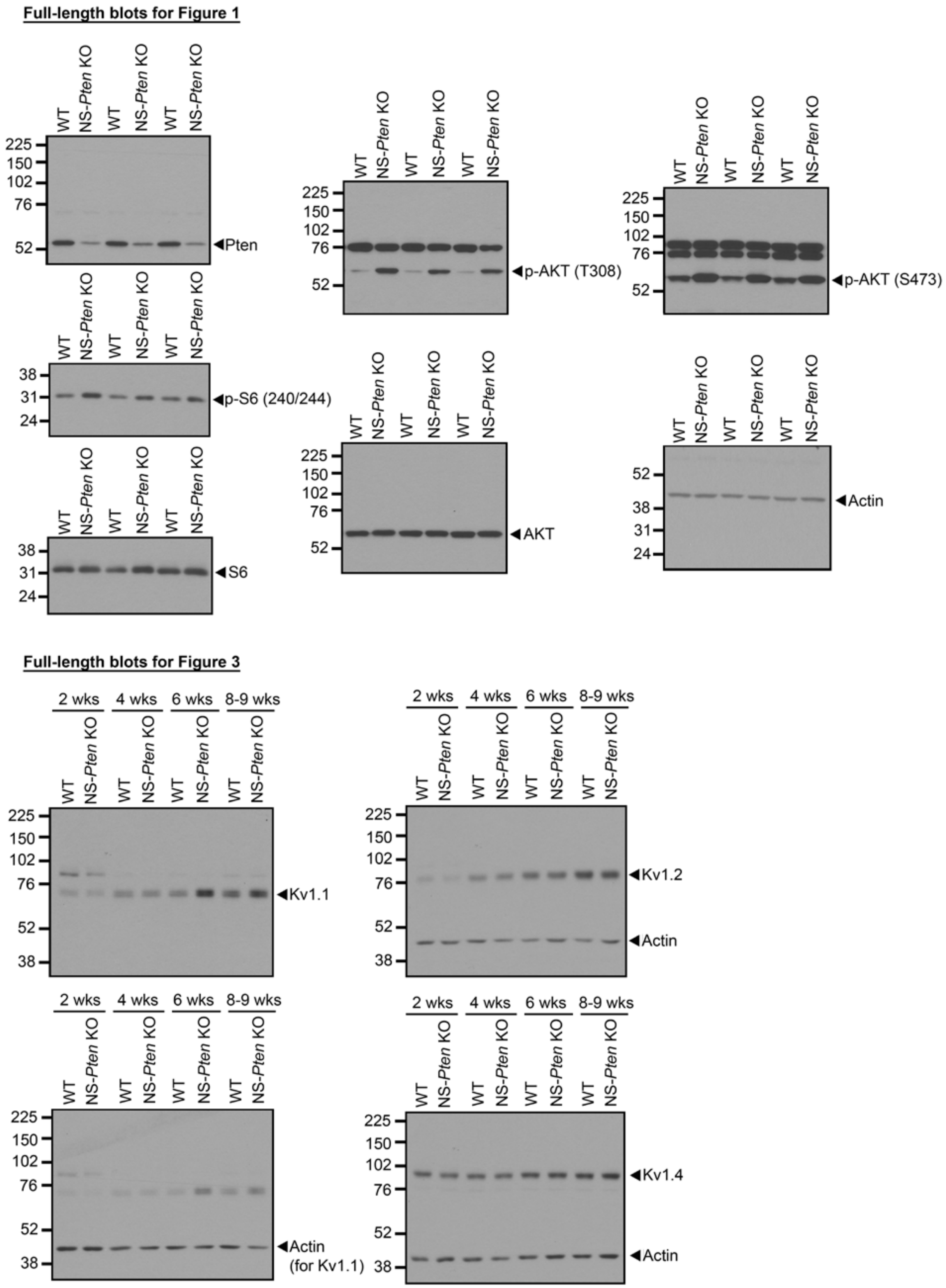

Supplemental figure 2: Full-length western blots.

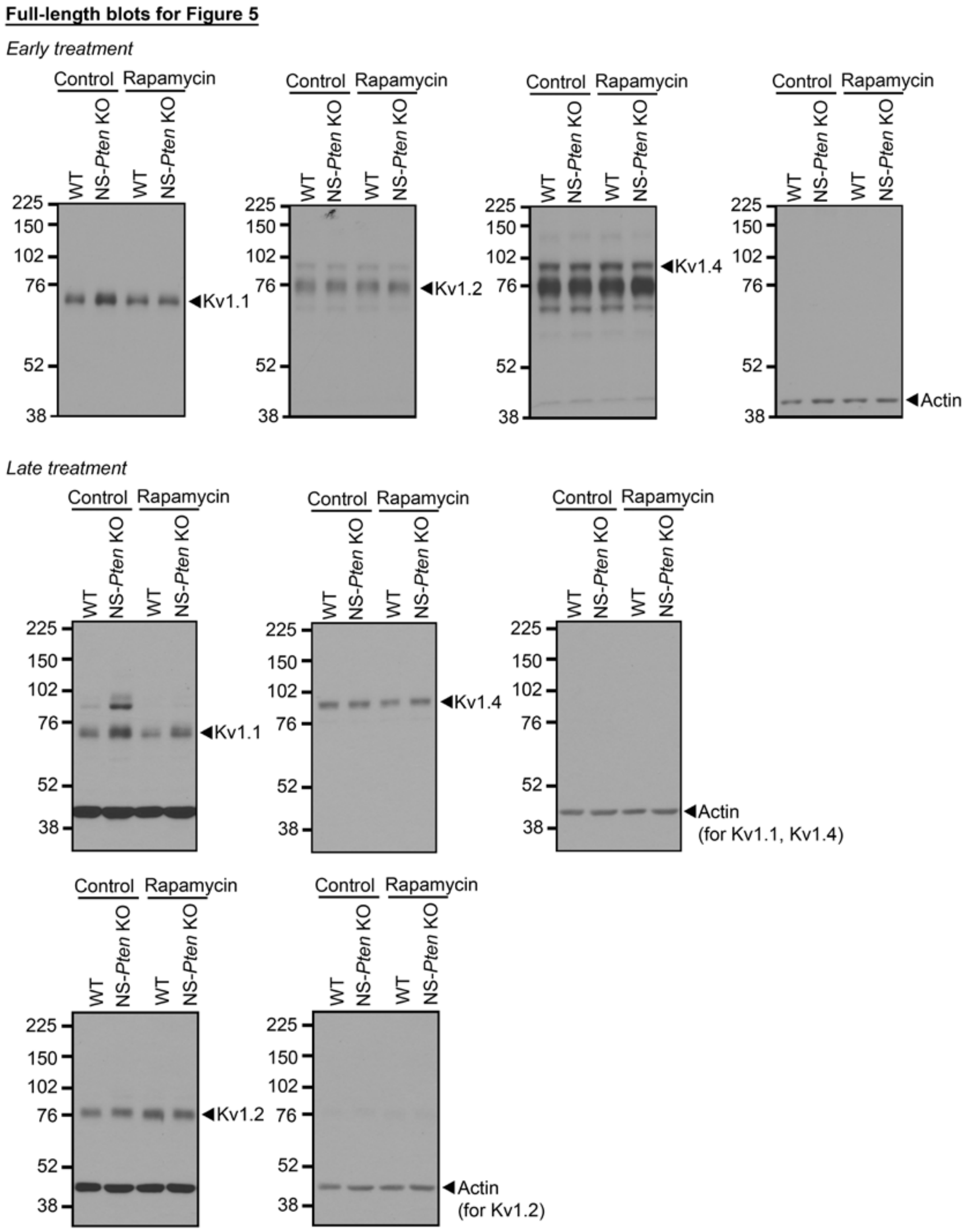

Supplemental figure 2: Full-length western blots (continued).
